# RETRACTION: Evaluation and Comparison of the Efficacy and Safety of the Combination of Topical Phenytoin and Microneedling With Microneedling Alone in the Treatment of Atrophic Acne Scars: A Controlled Blinded Randomized Clinical Trial

**DOI:** 10.1111/srt.70225

**Published:** 2025-09-14

Sadeghzadeh‐Bazargan A, Pashaei A, Ghassemi M, Dehghani A, Shafiei M, Goodarzi A. Evaluationand comparison of the efficacy and safety of the combination of topical phenytoin and microneedling with microneedling alone in the treatment of atrophic acne scars: A controlled blinded randomized clinical trial. *Skin Res Technol*. 2024;30(6):e13766. doi:10.1111/srt.13766

The above article, published online on 28 May 2024 in Wiley Online Library (wileyonlinelibrary.com), has been retracted by agreement between *Skin Research and Technology*; and John Wiley & Sons Ltd. The article was accepted for publication following an insufficient peer review process that does not meet the standards of the journal's peer review policy. Furthermore, scientific inconsistencies have been identified, and duplication has been detected within Figure 1. Accordingly, the article has been retracted. The authors have been informed of this decision. The corresponding author Azadeh Goodarzi agreed with the decision of retraction; the corresponding author Arezoo Pashaei disagreed with the decision of retraction; no confirmation was obtained by the remaining co‐authors.

